# Supplementary material for: Establishing Live-Cell Single-Molecule Localization Microscopy Imaging and Single-Particle Tracking in the Archaeon Haloferax volcanii
Source: Front Microbiol. 2020 Nov 20;11:583010. doi: 10.3389/fmicb.2020.583010 (PMC7714787; doi:10.3389/fmicb.2020.583010)
Supplement: Supplementary file 1 [file Data_Sheet_1.pdf]

Supplementary Information

Establishing live-cell single-molecule localization microscopy imaging and single-particle tracking in the archaeon *Haloferax volcanii*

Bartosz Turkowyd*[1], Sandra Schreiber*[2], Julia Wörtz[2], Ella Shtifman Segal[3], Moshe Mevarech[3], Iain G. Duggin[4], Anita Marchfelder#[2], Ulrike Endesfelder#[1,5]

- **Supplementary Text 1**
- **Supplementary Text 2**
- **Supplementary Figure 1**
- **Supplementary Figure 2**
- **Supplementary Figure 3**

Supplementary Text 1

Background sensitivity of single-molecule imaging

Fluorescence microscopy studies can be compromised by autofluorescence background. While conventional fluorescence microscopy in wild-type red Haloferax cells is generally possible as already a few GFP together provide sufficient signal above background, single-molecule imaging is severely compromised, especially at high frame rates of dynamic imaging.

The background level is an important factor in the detection and localization of single molecules. If signals are not sufficiently brighter than the background, a large proportion of the weaker signals will remain undetected, and brighter signals that are still visible above the background are localized at lower precision, directly limiting the overall resolution of the super-resolved image.

In our measurements, WR806 cells significantly improve single-molecule imaging conditions for *Haloferax*: **Supplementary Figure 2** shows that our fluorescent proteins yield on average ~ 200-250 photons per image (corresponding to 21.000 to 27.000 AD counts), typical numbers for single-molecule imaging. The pixel size is optimized for the detection and localization of single fluorescent spots and individual spots are measured on an area of about 3x3 pixels. Thus, the average signal of a single fluorescent protein is not much stronger than the average background of 1.300 AD counts per pixel in H119 cells (**Figure 1b**), which results a 9-pixel background signal of 11.700 AD counts (translates into ~ 107 photons for our setup). In contrast, WR806 cells are indistinguishable from the background outside cells and single fluorescent protein signals can be detected above background.

Supplementary Text 2

Sequences of fluorescent proteins

Dendra2 (*E. coli* opt)

ATGAACACCCCGGGTATTAACCTGATCAAAGAAGATATGCGCGTGAAGGTGCATATGGAGGGCA
ACGTGAATGGCCACGCCTTCGTGATCGAGGGCGAAGGTAAGGGCAAGCCGTATGAGGGCACCC
AGACAGCCAACCTGACCGTGAAAGAGGGCGCACCGCTGCCGTTCAGCTATGACATCCTGACCAC
AGCCGTGCACTACGGTAACCGCGTTTTCACCAAGTACCCGGAGGACATCCCGGACTACTTTAAG
CAGAGCTTCCCTGAGGGCTACAGCTGGGAGCGCACCATGACCTTTGAGGACAAGGGTATCTGCA
CCATCCGCAGCGACATCAGCCTGGAGGGCGACTGTTTCTTCCAGAACGTGCGCTTCAAGGGCAC
CAACTTCCCGCCGAATGGCCCGGTGATGCAGAAAAAGACCCTGAAGTGGGAGCCGAGCACCGA
GAAATTACACGTGCGTGACGGCCTGCTGGTGGGCAACATCAACATGGCCCTGCTGTTAGAGGGC
GGCGGCCACTATCTGTGCGACTTCAAGACCACCTACAAGGCCAAGAAGGTGGTGCAGCTGCCG
GACGCACACTTTGTGGACCACCGCATCGAAATCCTGGGCAACGACAGCGACTACAACAAGGTGA
AGTTATACGAGCACGCAGTTGCCCGCTATAGCCCGCTGCCTAGCCAGGTTTGGTAA

Dendra2Hfx

ATGAACACGCCGGGCATCAACCTCATCAAAGAGGACATGCGCGTCAAGGTCCACATGGAAGGCA
ACGTCAACGGCCACGCGTTCGTCATCGAAGGCGAAGGCAAGGGCAAGCCGTACGAAGGCACGC
AGACGGCGAACCTCACGGTCAAAGAAGGCGCCCCGCTCCCGTTCTCGTACGACATCCTCACGAC
GGCGGTCCACTACGGCAACCGCGTGTTCACGAAGTACCCCGAGGACATCCCGGACTACTTCAAG
CAGTCGTTCCCCGAGGGCTACTCGTGGGAGCGCACGATGACGTTCGAGGACAAGGGCATCTGC
ACGATCCGCTCGGACATCTCGCTCGAAGGCGACTGCTTCTTCCAGAACGTCCGCTTCAAGGGCA
CGAACTTCCCGCCGAACGGCCCGGTCATGCAGAAAAAGACGCTCAAGTGGGAGCCGTCGACCG
AGAAGCTCCACGTCCGCGACGGCCTGCTCGTCGGCAACATCAACATGGCGCTGCTCCTCGAAG
GCGGCGGCCACTACCTCTGCGACTTCAAGACGACGTACAAGGCGAAGAAGGTCGTCCAGCTCC
CGGACGCGCACTTCGTCGACCACCGAATCGAGATCCTCGGCAACGACTCGGACTACAACAAAGT
CAAGCTCTACGAGCACGCGGTCGCGCGATACTCGCCGCTCCCGTCGCAAGTCTGGTAA

```
Dendra2        1 ATGAACACCCCGGGTATTAACCTGATCAAAGAAGATATGCGCGTGAAGGTGCATATGGAG
Dendra2Hfx     1 ATGAACACGCCGGGCATCAACCTCATCAAAGAGGACATGCGCGTCAAGGTCCACATGGAA

Dendra2       61 GGCAACGTGAATGGCCACGCCTTCGTGATCGAGGGCGAAGGTAAGGGCAAGCCGTATGAG
Dendra2Hfx    61 GGCAACGTCAACGGCCACGCGTTCGTCATCGAAGGCGAAGGCAAGGGCAAGCCGTACGAA

Dendra2      121 GGCACCCAGACAGCCAACCTGACCGTGAAAGAGGGCGCACCGCTGCCGTTCAGCTATGAC
Dendra2Hfx   121 GGCACGCAGACGGCGAACCTCACGGTCAAAGAAGGCGCCCCGCTCCCGTTCTCGTACGAC

Dendra2      181 ATCCTGACCACAGCCGTGCACTACGGTAACCGCGTTTTCACCAAGTACCCGGAGGACATC
Dendra2Hfx   181 ATCCTCACGACGGCGGTCCACTACGGCAACCGCGTGTTCACGAAGTACCCCGAGGACATC

Dendra2      241 CCGGACTACTTTAAGCAGAGCTTCCCTGAGGGCTACAGCTGGGAGCGCACCATGACCTTT
Dendra2Hfx   241 CCGGACTACTTCAAGCAGTCGTTCCCCGAGGGCTACTCGTGGGAGCGCACGATGACGTTC

Dendra2      301 GAGGACAAGGGTATCTGCACCATCCGCAGCGACATCAGCCTGGAGGGCGACTGTTTCTTC
Dendra2Hfx   301 GAGGACAAGGGCATCTGCACGATCCGCTCGGACATCTCGCTCGAAGGCGACTGCTTCTTC

Dendra2      361 CAGAACGTGCGCTTCAAGGGCACCAACTTCCCGCCGAATGGCCCGGTGATGCAGAAAAAG
Dendra2Hfx   361 CAGAACGTCCGCTTCAAGGGCACGAACTTCCCGCCGAACGGCCCGGTCATGCAGAAAAAG

Dendra2      421 ACCCTGAAGTGGGAGCCGAGCACCGAGAAATTACACGTGCGTGACGGCCTGCTGGTGGGC
Dendra2Hfx   421 ACGCTCAAGTGGGAGCCGTCGACCGAGAAGCTCCACGTCCGCGACGGCCTGCTCGTCGGC

Dendra2      481 AACATCAACATGGCCCTGCTGTTAGAGGGCGGCGGCCACTATCTGTGCGACTTCAAGACC
Dendra2Hfx   481 AACATCAACATGGCGCTGCTCCTCGAAGGCGGCGGCCACTACCTCTGCGACTTCAAGACG

Dendra2      541 ACCTACAAGGCCAAGAAGGTGGTGCAGCTGCCGGACGCACACTTTGTGGACCACCGCATC
Dendra2Hfx   541 ACGTACAAGGCGAAGAAGGTCGTCCAGCTCCCGGACGCGCACTTCGTCGACCACCGAATC
```

```
Dendra2       601 GAAATCCTGGGCAACGACAGCGACTACAACAAGGTGAAGTTATACGAGCACGCAGTTGCC
Dendra2Hfx    601 GAGATCCTCGGCAACGACTCGGACTACAACAAAGTCAAGCTCTACGAGCACGCGGTCGCG

Dendra2       661 CGCTATAGCCCGCTGCCTAGCCAGGTTTGGTAA
Dendra2Hfx    661 CGATACTCGCCGCTCCCGTCGCAAGTCTGGTAA
```

PAmCherry1 (E. coli opt)

ATGGTAAGCAAAGGCGAAGAGGACAACATGGCGATCATTAAGGAGTTCATGCGCTTTAAGGTTCACAT
GGAGGGTAGCGTGAATGGTCATGTGTTTGAAATTGAGGGTGAGGGCGAGGGTCGTCCGTATGAAGGT
ACCCAAACCGCCAAACTGAAGGTCACGAAAGGTGGCCCGCTGCCGTTCACTTGGGACATCTTGAGCC
CGCAGTTCATGTACGGCTCCAACGCATACGTCAAACACCCAGCAGACATTCCGGACTACTTTAAACTG
AGCTTTCCGGAAGGCTTTAAGTGGGAGCGCGTTATGAAATTCGAAGATGGTGGTGTTGTCACCGTCAC
GCAGGACAGCAGCCTGCAGGATGGTGAGTTCATCTATAAAGTGAAACTGCGCGGTACCAACTTCCCG
TCTGATGGCCCGGTTATGCAAAAGAAAACCATGGGCTGGGAGGCACTGTCGGAGCGTATGTACCCGG
AAGATGGCGCGCTGAAAGGTGAGGTTAAGCCTCGTGTGAAACTGAAAGATGGCGGTCACTATGACGC
GGAAGTTAAGACGACCTATAAGGCTAAGAAGCCGGTCCAACTGCCGGGTGCGTACAATGTGAATCGT
AAGTTGGACATCACGAGCCATAACGAGGACTACACCATTGTGGAACAGTATGAGCGTGCCGAAGGTC
GTCACAGCACCGGTGGCATGGATGAACTGTACAAGTAA

PAmCherry1Hfx

ATGGTCAGCAAGGGCGAAGAGGACAACATGGCGATCATCAAAGAATTCATGCGCTTCAAGGTCCACA
TGGAAGGCTCGGTCAACGGCCACGTGTTCGAGATCGAGGGCGAGGGCGAAGGCCGACCGTACGAAG
GCACGCAGACGGCGAAGCTCAAAGTCACGAAGGGTGGCCCGCTCCCGTTCACGTGGGACATCCTCT
CGCCGCAGTTCATGTACGGCTCGAACGCGTACGTCAAGCACCCGGCGGACATCCCGGACTACTTCAA
GCTCTCGTTCCCCGAGGGCTTCAAGTGGGAGCGCGTCATGAAGTTCGAGGACGGCGGCGTCGTCAC
GGTCACGCAGGACTCGTCGCTGCAGGACGGCGAGTTCATCTACAAAGTCAAGCTCCGCGGCACGAA
CTTCCCGTCGGACGGCCCGGTCATGCAGAAAAAGACGATGGGCTGGGAGGCGCTCTCGGAGCGCAT
GTACCCCGAGGACGGGGCGCTCAAAGGCGAAGTCAAGCCGCGCGTCAAGCTCAAAGACGGCGGCCA
CTACGACGCGGAAGTCAAGACGACGTACAAGGCGAAGAAGCCGGTCCAGCTCCCCGGTGCCTACAA
CGTCAACCGCAAGCTCGACATCACGTCGCACAACGAGGACTACACGATCGTCGAGCAGTACGAGCGC
GCGGAAGGCCGACACTCGACGGGCGGCATGGACGAACTCTACAAATGA

```
PAmCherry1       1 ATGGTAAGCAAAGGCGAAGAGGACAACATGGCGATCATTAAGGAGTTCATGCGCTTTAAG
PAmCherry1Hfx    1 ATGGTCAGCAAGGGCGAAGAGGACAACATGGCGATCATCAAAGAATTCATGCGCTTCAAG

PAmCherry1      61 GTTCACATGGAGGGTAGCGTGAATGGTCATGTGTTTGAAATTGAGGGTGAGGGCGAGGGT
PAmCherry1Hfx   61 GTCCACATGGAAGGCTCGGTCAACGGCCACGTGTTCGAGATCGAGGGCGAGGGCGAAGGC

PAmCherry1     121 CGTCCGTATGAAGGTACCCAAACCGCCAAACTGAAGGTCACGAAAGGTGGCCCGCTGCCG
PAmCherry1Hfx  121 CGACCGTACGAAGGCACGCAGACGGCGAAGCTCAAAGTCACGAAGGGTGGCCCGCTCCCG

PAmCherry1     181 TTCACTTGGGACATCTTGAGCCCGCAGTTCATGTACGGCTCCAACGCATACGTCAAACAC
PAmCherry1Hfx  181 TTCACGTGGGACATCCTCTCGCCGCAGTTCATGTACGGCTCGAACGCGTACGTCAAGCAC

PAmCherry1     241 CCAGCAGACATTCCGGACTACTTTAAACTGAGCTTTCCGGAAGGCTTTAAGTGGGAGCGC
PAmCherry1Hfx  241 CCGGCGGACATCCCGGACTACTTCAAGCTCTCGTTCCCCGAGGGCTTCAAGTGGGAGCGC

PAmCherry1     301 GTTATGAAATTCGAAGATGGTGGTGTTGTCACCGTCACGCAGGACAGCAGCCTGCAGGAT
PAmCherry1Hfx  301 GTCATGAAGTTCGAGGACGGCGGCGTCGTCACGGTCACGCAGGACTCGTCGCTGCAGGAC

PAmCherry1     361 GGTGAGTTCATCTATAAAGTGAAACTGCGCGGTACCAACTTCCCGTCTGATGGCCCGGTT
PAmCherry1Hfx  361 GGCGAGTTCATCTACAAAGTCAAGCTCCGCGGCACGAACTTCCCGTCGGACGGCCCGGTC

PAmCherry1     421 ATGCAAAAGAAAACCATGGGCTGGGAGGCACTGTCGGAGCGTATGTACCCGGAAGATGGC
PAmCherry1Hfx  421 ATGCAGAAAAAGACGATGGGCTGGGAGGCGCTCTCGGAGCGCATGTACCCCGAGGACGGG

PAmCherry1     481 GCGCTGAAAGGTGAGGTTAAGCCTCGTGTGAAACTGAAAGATGGCGGTCACTATGACGCG
PAmCherry1Hfx  481 GCGCTCAAAGGCGAAGTCAAGCCGCGCGTCAAGCTCAAAGACGGCGGCCACTACGACGCG
```

```
PAmCherry1      541 GAAGTTAAGACGACCTATAAGGCTAAGAAGCCGGTCCAACTGCCGGGTGCGTACAATGTG
PAmCherry1Hfx   541 GAAGTCAAGACGACGTACAAGGCGAAGAAGCCGGTCCAGCTCCCCGGTGCCTACAACGTC

PAmCherry1      601 AATCGTAAGTTGGACATCACGAGCCATAACGAGGACTACACCATTGTGGAACAGTATGAG
PAmCherry1Hfx   601 AACCGCAAGCTCGACATCACGTCGCACAACGAGGACTACACGATCGTCGAGCAGTACGAG

PAmCherry1      661 CGTGCCGAAGGTCGTCACAGCACCGGTGGCATGGATGAACTGTACAAGTAA
PAmCherry1Hfx   661 CGCGCGGAAGGCCGACACTCGACGGGCGGCATGGACGAACTCTACAAATGA
```

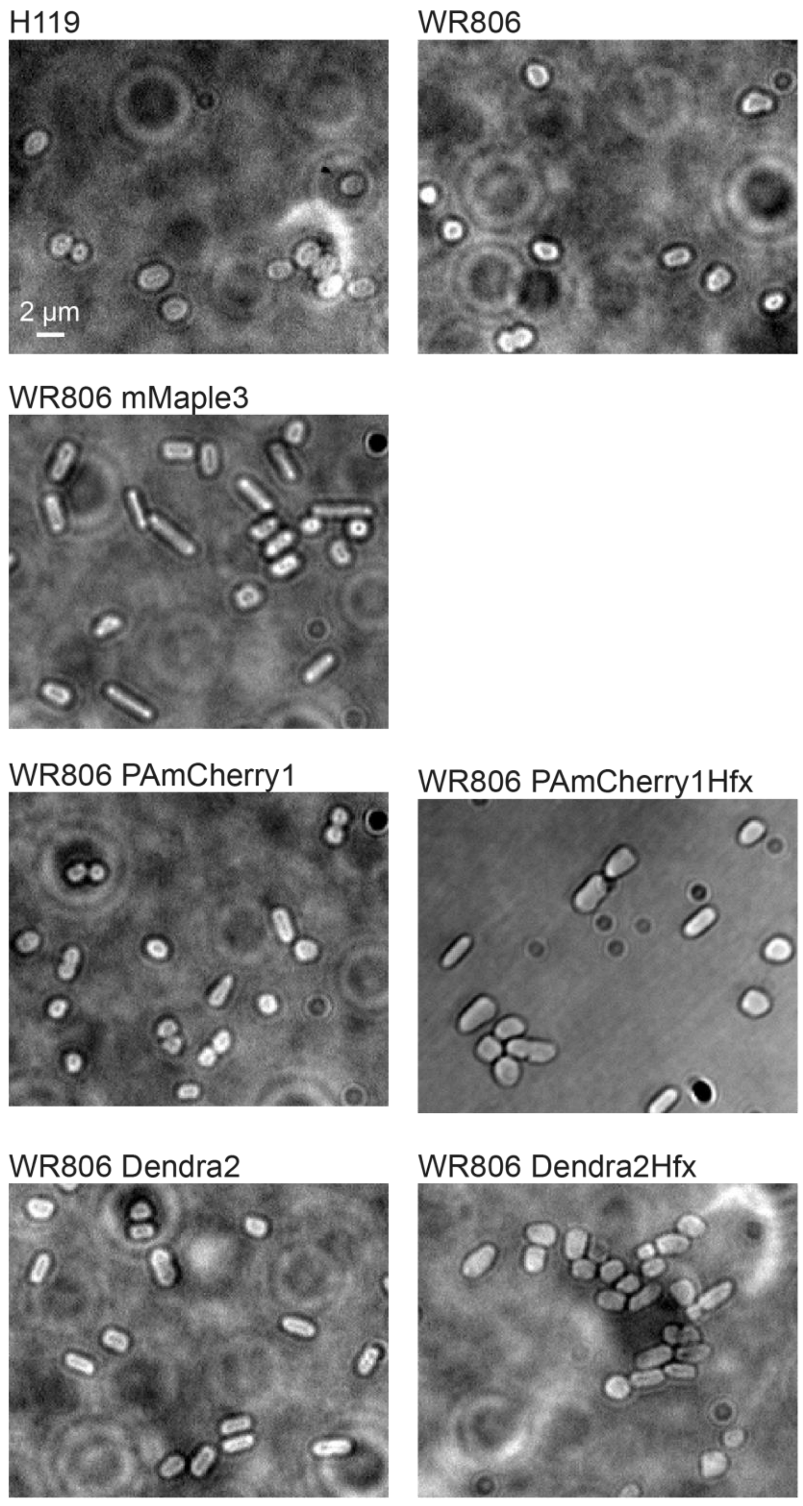

Supplementary Figure 1. Cell phenotype varies under the production of different fluorescent proteins

All strains producing fluorescent proteins which showed efficient photoactivation/-conversion (**Figure 1c**) showed similar phenotypes which are comparable to the parental strains H119 and WR806. In contrast, the strain producing mMaple3 shows strongly altered morphology. This and the fact that mMaple3 showed no fluorescence (**Figure 1c**) suggests improper protein folding that might cause increased cytotoxicity leading to a different growth phenotype.

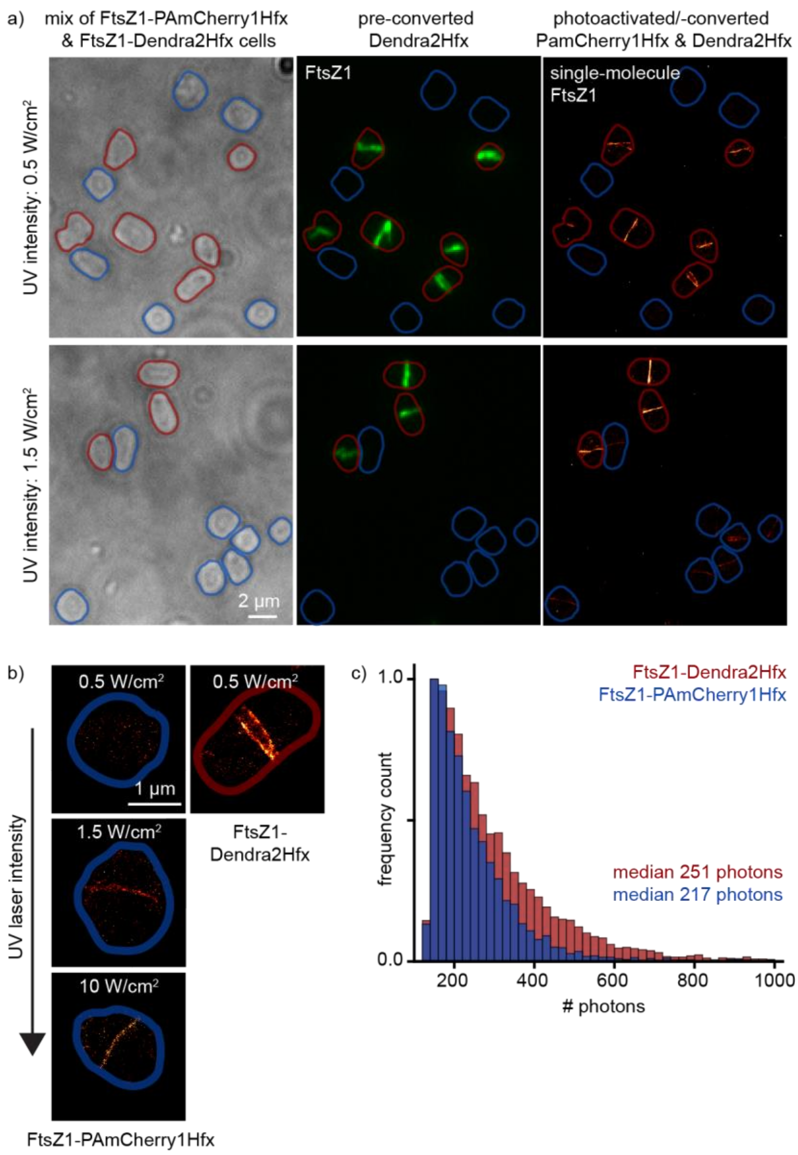

Supplementary Figure 2. Dendra2Hfx performs superior to PAmCherry1Hfx.
(a) WR806 cells producing either FtsZ1-Dendra2Hfx or FtsZ1-PAmCherry1Hfx were mixed on the same agarose pad and imaged under different UV laser intensities. When 0.5 W/cm² of the UV light was applied, FtsZ1-Dendra2Hfx revealed good photoconversion which results in dense SMLM ring structures. In contrast, FtsZ1-PAmCherry1Hfx was not photoactivated. A read-out of FtsZ1-PAmCherry1Hfx became only possible when increasing the UV light intensity to 1.5 W/cm².
(b) To provide an image quality comparable to FtsZ1-Dendra2Hfx when imaging FtsZ1-PAmCherry1Hfx, the UV light intensity had to be significantly increased. 10 W/cm² was barely sufficient to achieve the same quality as for the FtsZ1-Dendra2Hfx structure when applying 0.5W/cm².
(c) Under imaging conditions as in (a), Dendra2Hfx emits on average more photons (median of 251 photons) than PAmCherry1Hfx (median of 217 photons) per fluorescent emission in a single imaging frame.

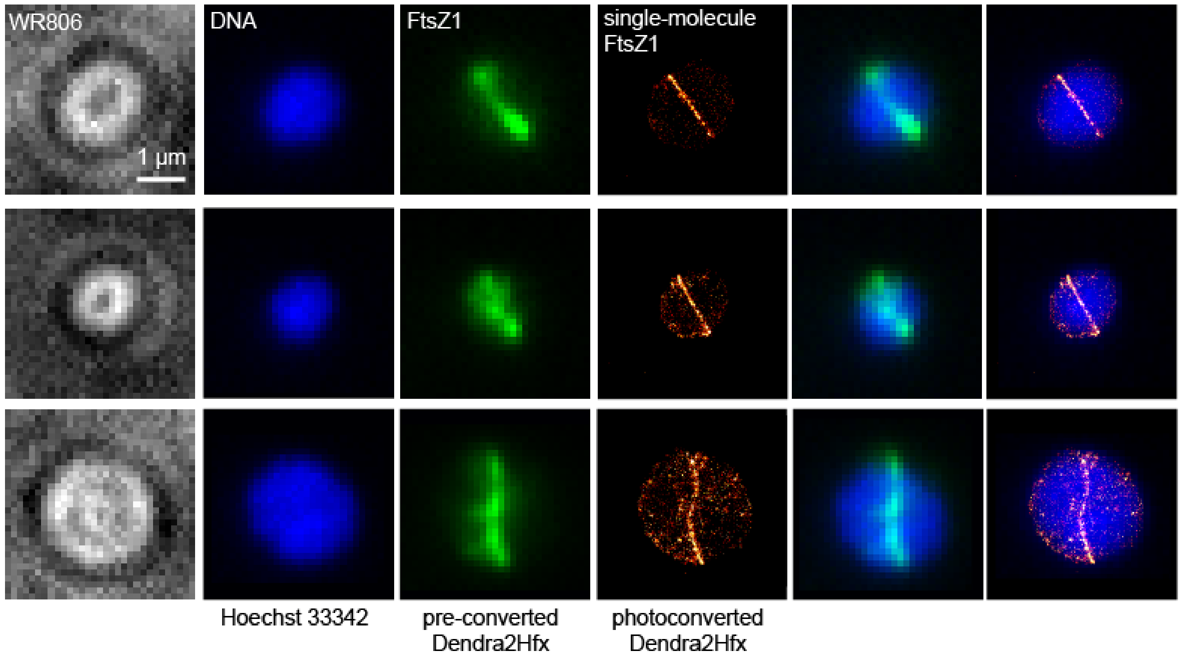

Supplementary figure 3. Dual color imaging of FtsZ1-Dendra2Hfx and DNA stained with Hoechst 33342. As FtsZ1-Dendra2Hfx can be photoconverted by primed photoconversion, which does not rely on UV light illumination, it is possible to use the DNA-intercalating dye Hoechst 33342 as a co-stain to obtain a DNA-reference image.
